# Diversity and Genetic Structure of Scarlet Plume (*Euphorbia fulgens*), an Endemic Plant of Mexico

**DOI:** 10.3390/plants11192542

**Published:** 2022-09-28

**Authors:** Mónica Pérez-Nicolás, Fabiola Ramírez-Corona, Teresa Colinas-León, Gisela Peña-Ortega, Ronald Ernesto Ontiveros-Capurata, Iran Alia-Tejacal, Fernando González-Andrés

**Affiliations:** 1 Departamento de Fitotecnia, Universidad Autónoma Chapingo, Texcoco 56230, Mexico; 2 Taller de Sistemática y Biogeografía, Departamento de Biología Evolutiva, Facultad de Ciencias de la UNAM, Ciudad de México 04510, Mexico; 3 Coordinación de Riego y Drenaje, Instituto Mexicano de Tecnología del Agua (IMTA), Jiutepec 62550, Mexico; 4 Facultad de Ciencias Agropecuarias, Universidad Autónoma del Estado de Morelos, Cuernavaca 62210, Mexico; 5 Instituto de Medio Ambiente, Recusos Naturales y Biodiversidad, Universidad de León, 24009 León, Spain

**Keywords:** microendemic species, distribution, multi-criteria analysis, Oaxaca

## Abstract

*Euphorbia fulgens* is an ornamental species cultivated in Europe and endemic to Mexico; its ecological, genetic, and evolutionary aspects are not known. The objectives of this study were to determine its distribution, describe the places it inhabits, and analyze the diversity and genetic structures of wild populations of *E. fulgens*. A bibliographic review of the herbarium specimens and a field evaluation were carried out to develop a potential distribution map based on a multi-criteria analysis of the climatic and topographic variables. Three populations (forty-five individuals) from pine–oak and cloud forests located in the Southern Sierra of Oaxaca were analyzed using ten microsatellite loci. The analysis was conducted using Arlequin v. 3.5, Mega v. 10, and Structure v. 2.3 programs. Eight loci were polymorphic, and a total of thirty-eight alleles were obtained. The average number of alleles per polymorphic locus was 4.6. The average heterozygosity of the three populations was high (Ho = 0.5483), and genetic differentiation between populations were low, with a high genetic flow, suggesting that it could be an ancestral population that became fragmented and was just beginning to differentiate genetically. The information generated on this restricted distribution species can be used in conservation programs pertaining to human activities that endanger the habitats where it is found.

## 1. Introduction

The family Euphorbiaceae is represented by 8000 to 9000 species [1,2], making it one of the most important within the angiosperms. Current phylogenetic and molecular studies have changed their taxonomies into five families: Phyllanthaceae, Picrodendraceae, Peraceae, Putranjivaceae, and Euphorbiaceae [3]. In Mexico, it is one of the most diverse families and is composed of 714 species. *Euphorbia* is a diverse genus with 245 species, of which, 132 are endemic [4]. However, other authors report 114 endemic species out of a total of 241 species [5] and 81 endemic species out of a total of 138 species [6]. There are 38 species localized in one state of the country [4], among them, *Euphorbia fulgens* Karw. ex Klotzsch belongs to the subgenus *Chamaesyce* sect. *Alectoroctonum* [7].

*Euphorbia fulgens* is found in three localities in the state of Oaxaca; it is collected from wild populations and is used for religious purposes. In other countries, ornamental cultivated varieties are sold mainly as cut flowers and potted plants. It is a phytogenetic resource that can be sustainably exploited in Mexico. Since it has aesthetic values and adapts easily to cultivation conditions, recently, differences in morphological vegetative and reproductive structures were found in wild and cultivated populations [8].

However, it has been studied very little and the ecological, evolutionary, diversity, and genetic structural aspects that provide information on its endemism are unknown. Studies on the genetic diversities and structures using microsatellites help estimate the genetic variations in wild populations, especially in species of restricted and endemic distributions. Knowing whether populations are genetically differentiated at different levels makes it possible to identify the environmental factors that affect them. Estimating the genetic flow between populations, in turn, helps to determine the levels of inbreeding or exogamy, which is convenient for the management and conservation of plant genetic resources [9,10,11].

There are few studies on the genetic diversity of the genus *Euphorbia*; abundant genetic variations in wild populations of *Euphorbia pulcherrima* in several localities have been found in Mexico via plastid (trnG-trnS, psbA-trnH) and nuclear (G3pdh) regions [12]. In China, *Euphorbia kansui*, endemic to this country, was molecularly characterized using 12 microsatellites. The number of alleles ranged from 2 to 11, with an average of 4.1 alleles per locus [13]. In a study on *Euphorbia palustris*, a species endemic to Europe, seven microsatellites were used to study the genetic structures and gene flow among its populations; between 3 and 13 alleles per locus were found, as well as high levels of heterozygosity [14]. Additionally, populations of *Euphorbia lamarckii*, a species endemic to the Canary Islands, was molecularly characterized, using 10 microsatellite loci, between 2 and 13 alleles per locus were found [15].

Molecular studies using microsatellites in plant species belonging to other families with restricted distributions were useful to evaluate the genetic differentiation between populations. Thus, using ten microsatellites in populations of *Guaiacum unijugum*, it was found that genetic differentiations among them were reduced and attributed to the presence of rare and unique alleles [16]. In *Paeonia jishanensis*, endemic to China, genetic diversity and population structures were assessed using 21 microsatellite loci, and moderate levels of genetic diversity were detected [17]. In the populations of *Petunia secreta*, a rare species endemic to Brazil, 15 microsatellite loci found high genetic diversity among them [18]. The study of the populations of *Sophora alopecuroides*, a species endemic to China, 18 microsatellite loci allowed researchers to estimate a low genetic diversity based on the number of effective alleles; such genetic variabilities were higher within populations than among them [19].

Therefore, the present study is the first of its kind for the genus *Euphorbia*, with species that are endemic to Mexico. The objectives were to define the geographic distribution, describe the habitat, and determine the genetic diversity and structure of wild populations of *Euphorbia fulgens*, which are so far unknown. It will provide valuable information for the management and preservation of this species. Considering that this species presents a restricted distribution and is endemic to Mexico, it must have low genetic diversity and genetic flow.

## 2. Results

### 2.1. Geographical Distribution

*Euphorbia fulgens* is only distributed in the State of Oaxaca. So far, only three populations have been documented: (a) in the municipality of San Jerónimo Coatlán; (b) on the borders of the municipalities of Santiago Jamiltepec and Santiago Tetepec, 80 km in a straight line from (a); and (c) in the municipality of Santa Catarina Juquila, 50 km from (b), so this species is considered endemic to Sierra Sur de Oaxaca.

**Figure 1:**
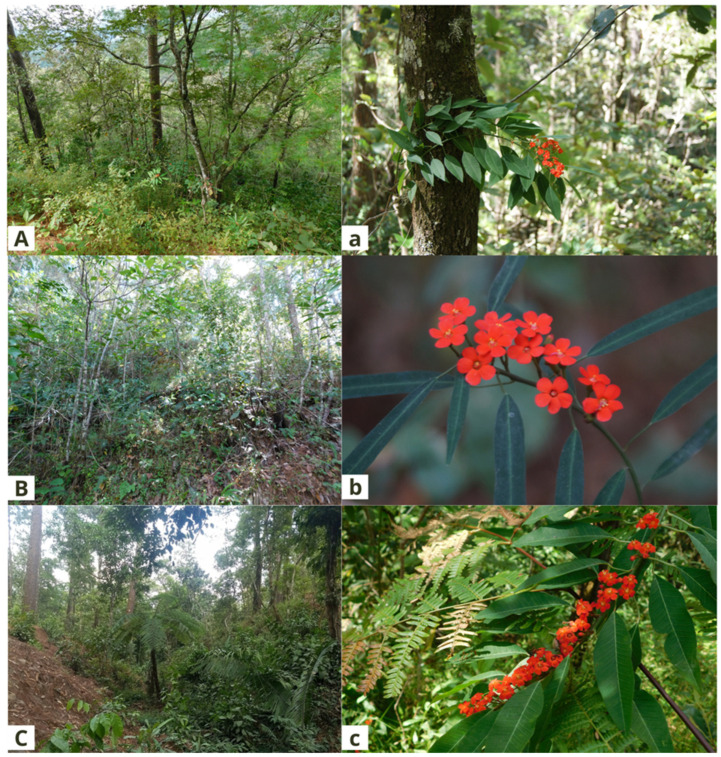
Wild populations of *Euphorbia fulgens* in the state of Oaxaca. Population 1, (**A**,**a**). San Jerónimo Coatlán; population 2 (**B**,**b**). Santiago Jamiltepec; population 3 (**C**,**c**). Santa Catarina Juquila. The capital letters show the species’ habitat, and the lowercase letters show its inflorescences.

The populations are in canyons with steep slopes that are difficult to access; in areas with pine–oak forests (POFs); a combination of pine–oak forests and cloud forests (POF-CFs); cloud forests (CFs), and the secondary vegetation derived from the above. Population 1 is located at 1421 m in a POF; population 2 is located at 1160 m in a POF-CF and CF zone; population 3 is located at 1130 m in a CF and the secondary vegetation derived from it (Figure 1).

Population 1 is located in places that are difficult to access, with steep slopes that are far from human populations, so there is little disturbance (although habitat alterations were observed due to the opening of roads for timber extraction). Population 2 is located on steep slopes, with habitat alterations due to agricultural and cattle-ranching activities. Finally, population 3 is in conserved areas with secondary vegetation derived from CFs. In the latter case, there are disturbances due to the proximity of human settlements, so the survival of this species is at risk.

**Figure 2:**
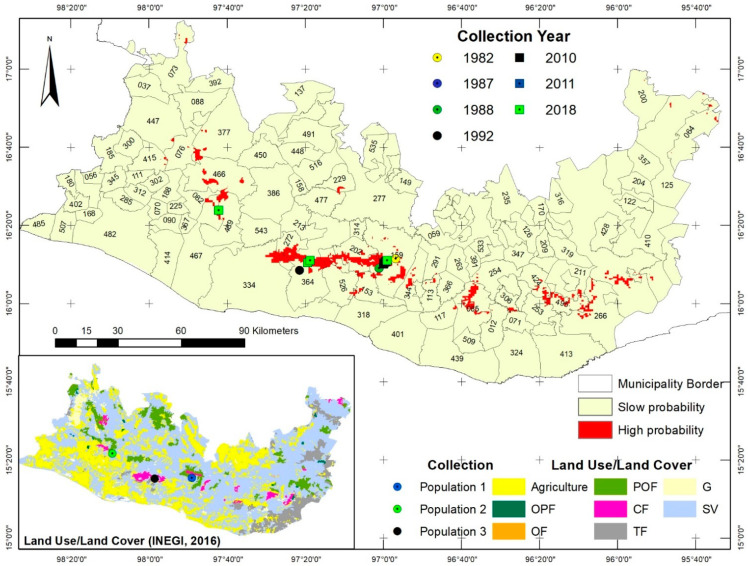
Potential distribution of *Euphorbia fulgens* in the Sierra Sur and Costa regions of Oaxaca.

The analysis of climatic variables (temperature and precipitation), topographic variables (altitude and slope), and vegetation type at the collection points showed that the species is distributed within the ranges of precipitation, temperature, slope, altitude, and vegetation type indicated in Table 1. Furthermore, the multi-criteria analysis results indicate that the type of vegetation/land use–land cover where the species is found seems to be the most discriminating variable as it corresponds to 25% of the relative area, indicating that the species has a potential area of 54,665 ha (Figure 2).

The analysis of the soil samples showed that the sites have similar characteristics. They are sandy–loam textured soils, with pH values 5.0, EC < 0.06 dS m^−1^, and a bulk densities (BD) of 1.0 g cm^−3^. The soil of population 1 contained 53.3% sand, 29.3% silt, and 17.2% clay. The soil of population 2 presented 55.5% sand, 31.3% silt, and 13.2% clay, while the soil of population 3 contained 65.9% sand, 16.0% silt, and 18.1% clay. The soil of population 1 had the lowest organic matter content (4.48%). The soil of population 2 had higher Ca (910 mg kg^−1^), Mg (227 mg kg^−1^), Fe (110 mg kg^−1^), Zn (1.28 mg kg^−1^), and Mn (14.56 mg kg^−1^) contents compared to the other populations (Table 2).

The *Euphorbia fulgens* plants collected at the three sites have formed independent populations because they are separated by roads, highways, and human settlements. Therefore, no studies on reproduction and pollination have been carried out and the chromosomic number is not known. However, field observations in this study suggest that it could be an allogamous species with entomophilous pollination, mainly by Diptera, tiny wasps, bugs, and ants.

### 2.2. Molecular Characterization

Only 8 of the 10 microsatellites used were amplified; all were polymorphic and generated 38 alleles. The markers with the highest alleles were Ep90 (12 alleles), Ep05 (6 alleles), E97, Ep61 (4 alleles), and the rest with 3 alleles. The average number of alleles per polymorphic locus was 4.6, so allelic diversity was relatively low to moderate. Total heterozygosity (HT) for the three analyzed populations of *E. fulgens* was high (0.5483). The analysis at the intra-population level indicated that in the eight loci, the average observed heterozygosity in population 1 was 0.6468, in population 2, it was 0.5835, and in population 3, it was 0.4148. Population 1 showed higher genetic diversity by observed heterozygosity and the number of observed genotypes (Go = 114) compared to populations 2 and 3 (Table 3), indicating that, although these eight microsatellite loci are not specific to *E. fulgens*, they can be used to evaluate this species structure and genetic diversity.

The overall molecular analysis of variance (AMOVA) using the eight amplified loci showed that the most significant proportion of molecular variability was located among individuals (93.84%) and not among populations (5.58%). Wright’s F-statistics for measuring genetic structure showed low values of genetic differentiation between populations (FST = 0.05587) and low levels of total inbreeding in the three populations (FIS = 0.00602 and FIT = 0.06156). Although there were missing data when we used all eight loci, AMOVA was performed with the three most representative loci (1, 2, and 7). The results indicate that 5.57% of the genetic variations are between populations, 6.55% are in individuals within populations, and 87.88% are within individuals of the three populations (Table 4). Wright’s F-statistics are similar to those obtained for the eight microsatellite loci; genetic differentiation is low, with little or no intrapopulation subdivision (FST = 0.05597) and low levels of total inbreeding (FIS = 0.06936 and FIT = 0.12117). The population-specific FIS indices show no inbreeding; even in population 1, it indicates exogamy, which would explain that there is no intrapopulation subdivision. The population-specific FIS indices (Table 5) show no inbreeding, even in population 1, indicating exogamy, which would explain that there is no intrapopulation subdivision.

**Figure 3:**
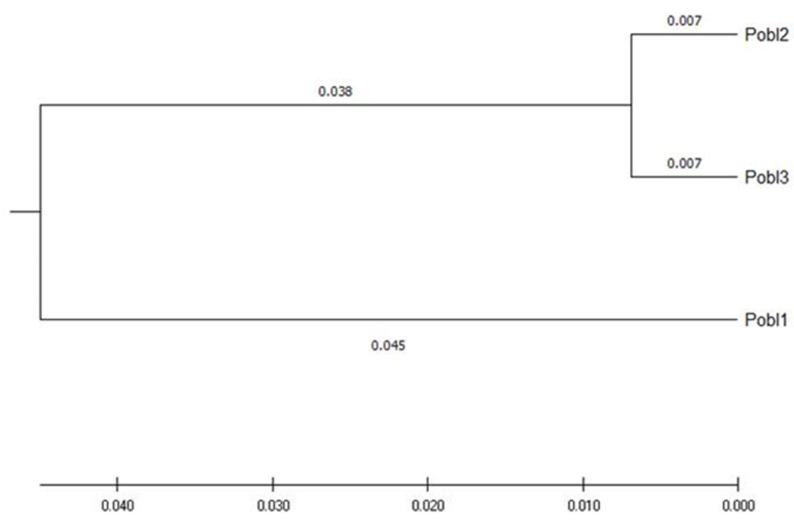
UPGMA tree of three populations, distance method: number of different FST alleles.

A pairwise comparison of the populations using paired *t*-tests indicates that population 1 differs from populations 2 and 3. However, the latter does not differ (Table 6), which is consistent with the unweighted pair group method with arithmetic mean (UPGMA) analysis, where populations 2 and 3 are grouped on one side, and population 1 is separated (Figure 3). In addition, the level of gene flow between the three populations was high, especially between populations 2 and 3 (Table 7).

**Figure 4:**
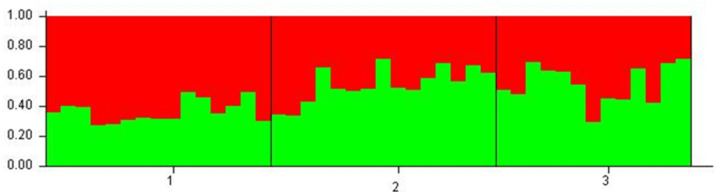
Analysis of the genetic structure of three populations of *Euphorbia fulgens* with eight SSRs. Graph generated with the outputs of the Structure software and organized by the K value. Each color represents a cluster defined by the software for populations 1, 2, and 3.

The Bayesian structuring analysis showed that the populations were divided into two large genetic groups or clusters (K = 2) (Table 8). The expected heterozygosity among individuals in the same cluster was 0.6115 for cluster 1 and 0.5316 for cluster 2, indicating high genetic variation in both clusters. The three populations have mixed distributions (Figure 4); most individuals from population 1 were grouped in cluster 1, while individuals from populations 2 and 3 were distributed similarly in both clusters. The fixation index obtained for cluster 1 (FST = 0.0262) was lower than that of cluster 2 (FST = 0.1739), suggesting a higher degree of genetic differentiation among individuals in this second group, although this difference was not significant. The estimated gene flow value in group 1 (Nm = 9.29) was much higher than in group 2 (Nm = 1.1876), suggesting no genetic differentiation due to the high gene flow by cross-pollination or allogamy.

## 3. Discussion

The total heterozygosity for the three populations of *Euphorbia fulgens* (HT = 0.5483) indicated a high genetic diversity despite its endemic and restricted distributions. Population 1 had the highest genetic diversity and was separated from populations 2 and 3. However, this difference was not significant according to the Bayesian analysis conducted with Structure software. The levels of genetic diversity in *E.* *fulgens* are similar to those obtained in *E. kansui*, a species endemic to China [13], *E. lamarckii*, a species endemic to the Canary Islands [15], and *E. palustris*, with a peculiar distribution limited to the banks of rivers in Central Europe [14]. Similarly, moderate and high variation levels have been documented in endemic species that do not belong to the genus *Euphorbia*, such as *Pityopsis ruthii* [20], *Seseli farrenyi* [21], and *Aristolochia delavayi* [22]. However, it has been suggested that species with a wide distribution, cross-reproduction, wind pollination, and long generation times show more significant variations than those with the opposite characteristics, as with *Eriocaulon bilobatum*, an aquatic species with sexual and asexual reproduction, monoecious, and that is pollinated by insects with isolated and scarce populations [23]. Although *Lilaea scilloides* is widely distributed, it has shown low genetic diversity within and among the populations analyzed [24].

The genetic structure analyzed for *E. fulgens* showed that the highest proportion of molecular variability was found within individuals rather than between populations. There were low values of genetic differentiation between populations and low levels of total inbreeding in the three populations; the same occurs in endemic species, such as *Paeonia decomposita*, where the highest proportion of molecular variability is found within populations [25]. This has also been observed in species with few populations with low population differentiation [23] or in species distributed along rivers, such as *Aristolochia delavayi* [22]. A pairwise comparison of populations using paired *t*-tests indicated that population 1 differed from populations 2 and 3, but the latter did not differ. The gene flow level between the three populations was high, especially between populations 2 and 3. This suggests that this is an allogamous species with pollination mainly by dipterans, tiny wasps, bugs, and ants observed in the inflorescences during fieldwork. However, no studies have documented the pollinators of *E. fulgens*, so it is proposed that researchers conduct studies to verify the pollination and reproduction systems of this species of *Euphorbia* and, thus, complement the factors that contribute to the high gene flow levels.

It should be noted that the populations are not very far from each other; the distance between populations is less than 100 km in a straight line. Populations 2 and 3 are in a cloud forest and ecotone 1 (POF-CF) inhabited areas. In contrast, population 1 inhabits a pine–oak forest and ecotone 1 (POF-CF), but at a higher altitude than the other populations and with difficult access, which also explains why it is the population with the most significant genetic variation and slight differentiation; however, the gene flow between the three populations is still being maintained. It agrees with what was obtained via the Bayesian analysis of genetic structuring using Structure software, which indicated that the analyzed populations were divided into two large genetic groups or clusters (K = 2), and that most of the individuals of population 1 were grouped in group 1 and the remaining (populations 2 and 3) in group 2. However, there is a mixed population with a slight genetic differentiation between individuals of group 2 and individuals of group 1 (without being significant). The high gene flow values suggest a lower genetic differentiation due to the entry of genes related to cross-pollination or allogamy; the same is documented in *Seseli farrenyi*, an endemic species with a minimal distribution range in the Mediterranean Basin [21]. Therefore, it could well be a large population that inhabited a large geographic area of a cloud forest or oak–pine forest, showing a sign of fragmentation due to multiple factors, e.g., biological–evolutionary processes, such as genetic drift, natural selection, and migration, or by human activities and changes in the climatic conditions of that region.

## 4. Materials and Methods

### 4.1. Geographical Distribution

The geographic distribution information of *Euphorbia fulgens* was obtained from the databases of national and international open access collections available on the internet January 2018: Global Biodiversity Information Facility (www.gbif.org, accessed on 22 September 2022), Red Mundial de Información Sobre Biodiversidad (REMIB) (http://www.conabio.gob.mx/remib, accessed on 22 September 2022), the open data website of the Universidad Nacional Autónoma de México (UNAM) (http://datosabiertos.unam.mx, accessed on 22 September 2022), and the Tropicos database of the Missouri Botanical Garden (www.tropicos.org, accessed on 22 September 2022). In addition, information was collected from herbarium specimens deposited in the most important collections in Mexico (MEXU, ENCB, IEB). A database was constructed containing the state, municipality, locality, latitude, longitude, type of vegetation, altitude, collector number, collector, name of the identifier, and herbarium of where it was located, with the records of the herbarium specimens. Additionally, specialists in the family Euphorbiaceae were consulted on the geographic distribution of the species in Mexico.

Data on altitude, vegetation type, and geographic coordinates were taken between December 2018 and January 2019 at the collection sites. In addition, in order to have physical and chemical characterizations of the soil, three samples of one kilogram were taken at a depth of 20 cm and analyzed to obtain the pH, electrical conductivity (dS m^−1^), percentage of organic matter, inorganic nitrogen (mg kg^−1^), phosphorus (mg kg^−1^), potassium (mg kg^−1^), calcium (mg kg^−1^), magnesium (mg kg^−1^), iron (mg kg^−1^), copper (mg kg^−1^), zinc (mg kg^−1^), manganese (mg kg^−1^), boron (mg kg^−1^), bulk density (g cm^−3^), and texture (percentage of sand, silt, and clay).

Based on the information obtained, geographic and potential distribution maps were prepared via a multi-criteria analysis using QGIS software v. 3.10. The variables considered were climate (climatic units, temperature, and precipitation), terrain slope, altitude, and vegetation type obtained from the UNAM database and the Instituto Nacional de Estadística y Geografía [26] (Table 9).

### 4.2. Molecular Characterization

Molecular characterization was performed on 45 individuals from 3 populations. Youngs, healthy leaves were collected and packaged in Ziploc^®^ bags and transported under refrigerated conditions to the laboratory for analysis. DNA was extracted using the CTAB methodology [27] with some modifications. The quality of purity and the quantity of DNA (ng/µL^−1^) were determined using a Thermo Scientific^®^ (MA, USA) brand Nanodrop Lite, for which all samples were diluted to 10 ng/µL^−1^. DNA quality was evaluated by 2% agarose gel electrophoresis at 120 volts for one and a half hours, which was subsequently stained with ethidium bromide and documented with low-cost image acquisition and documentation of gels, Digidoc-lt^®^ (NJ, USA) Imaging System.

Since there were no previous molecular characterization studies of *Euphorbia fulgens*, in the present study, ten microsatellite molecular markers used in other species of the same genus, *E. kansui*, endemic to China [13], *E. lamarckii*, endemic to the Canary Islands [15], and *E. palustris*, native to Europe [14], were used. PCR was performed for all ten microsatellite pairs without fluorescent labeling (Supplementary Material Table S1). The PCR reaction mixture had a final volume of 15.6 µL, consisting of: 9.45 µL of molecular biology grade water, 3.00 µL of 5X reaction buffer (Bioline^®^, Meridian Life Science Inc. USA), 0.5 µL of forwarding primer (T4Oligo^®^), 0.5 µL of reverse primer (T4Oligo^®^), 0.15 µL of Taq polymerase (Bioline^®^ 500 U), and 2.00 µL of DNA. Amplification was performed with Techne Flexigene^®^ (CB, UK) thermal cyclers with the following PCR sequence: 1 pre-denaturation cycle at 94 °C for 5 min, 35 denaturation cycles at 94 °C for 30 s, 1 alignment cycle of 30 s at the optimal recommended temperature for each primer pair, 35 extension cycles at 72 °C for 30 s, and 1 final extension cycle at 72 °C for 5 min. The amplified fragments were separated by electrophoresis at 110 volts for 40 min on 3.5% MetaPhor^®^-Nusieve^®^ (ME, USA) agarose gels and 1X TBE buffer. Gels were developed using a Red Safe^TM^ (NJ, USA) staining solution; the gels were documented with a Labnet^®^ (NC, USA) ultraviolet light transilluminator.

### 4.3. Statistical Analysis

The following statistical tests were performed from the banding patterns: Hardy–Weinberg, analysis of molecular variance (AMOVA), average Wright’s F-statistics, and an exact test of the population differentiation for the population pairs. From these tests, and using Arlequin v. 3.5 [28], statistics of the expected and observed heterozygosity, genetic diversity within and between populations, and level of gene flow, were obtained. In addition, calculated Fst data were used to generate a genetic distance matrix, and a UPGMA analysis was performed using Mega version X software [29].

On the other hand, estimates of the most probable numbers of populations (K) were obtained using the Structure program, version 2.3 [30]. The program was run with 6 independent replicates for K (i.e., distinct populations or gene clusters with K from one to 10) with a discard period of 10,000 replicates followed by 50,000 Monte Carlo simulations in a model assuming no admixtures and independent allele frequencies. Finally, the most probable number of clusters was determined using the method proposed by Evanno et al. [31].

## 5. Conclusions

*Euphorbia fulgens* has a distribution restricted to a single state; three geographically separated populations were located, making it endemic to the Sierra Sur region of Oaxaca. Genetic diversity was high without significant genetic differentiation, and the molecular genetic structure revealed that there are only two genetic populations instead of the three that had been defined a priori based on their geographic locations; these populations maintain a high level of gene flow and prevent genetic divergence. Genetic differentiation between populations 2 and 3 is very low; both likely came from population 1, which is fragmented and just beginning to differentiate genetically. This study provides information that can be used in conservation programs due to the risk of extinction related to habitat fragmentation and shows the need for further ecological–evolutionary studies, to determine the pollination systems of these plants, to know their pollinators, and elucidate their evolutionary history to explain their endemism.

## Figures and Tables

**Table 1:** Multi-criteria analysis for the potential distribution of *Euphorbia fulgens*.

Variable	Min	Max	Area (ha)	% *
Precipitation (mm)	1032	1824	1,992,762	2.7
Temp. min (°C)	11.5	15	537,273	6.7
Temp. max (°C)	26	29	819,153	10.2
Slope (%)	7	17	1,103,041.53	4.9
Altitude (m)	1129	1670	1,159,670	4.7
Vegetation type	Pine–oak forest (BOF) Cloud forest (CF) Shrub and arboreal secondary vegetation of CF		218,743.65	25.0
Potential distribution			54,665.19	

* The relative proportion of the potential surface area of each variable.

**Table 2:** The physical and chemical characteristics of soil in three populations of *Euphorbia fulgens* in the state of Oaxaca, Mexico. 1. San Jerónimo Coatlán. 2. Santiago Jamiltepec. 3. Santa Catarina Juquila *.

**Population**	**Texture**			**pH**	**EC** **dS m^−1^**	**MO** **%**	**N Inorg.**	**P** **mg kg^−1^**	**K** **mg kg^−1^**	**Ca** **mg kg^−1^**
	**S %**	**L %**	**C %**							
**1**	53.3	29.3	17.2	5.27	0.03	4.48	11.4	0.78	230	287
**2**	55.5	31.3	13.2	5.43	0.06	7.07	15.9	2.77	364	910
**3**	65.9	16.0	18.1	5.25	0.04	7.54	15.8	4.73	144	164
**Population**				**DBD** **g cm^−3^**	**Mg** **mg kg^−1^**	**Fe** **mg kg^−1^**	**Cu** **mg kg^−1^**	**Zn** **mg kg^−1^**	**Mn** **mg kg^−1^**	**B** **mg kg^−1^**
**1**				1.20	98	35.61	0.56	0.40	2.52	1.63
**2**				1.10	227	110.54	0.40	1.28	14.56	1.98
**3**				1.11	43	63.21	0.12	0.60	5.98	1.58

EC: Electrical conductivity. dSm^−1^: deciSiemens per meter. EC: Electrical conductivity. MO: Organic matter. DBD: Bulk density. * See methodology analysis in the supplementary material S2.

**Table 3:** Measures of genetic diversity of three populations of *Euphorbia fulgens*.

Locus	Population 1				Population 2				Population 3			
	Go	Ho	He	*p*-Value	Go	Ho	He	*p*-Value	Go	Ho	He	*p*-Value
Ek3216	14	0.28571	0.75132	0.00010	15	0.40000	0.61609	0.01014	13	0.15385	0.60000	0.00036
Ek8578	15	0.93333	0.63678	0.07935	15	0.73333	0.71954	0.09874	13	0.92308	0.68615	0.01717
E78	13	0.84615	0.50769	0.02360	11	0.81818	0.50649	0.06619	7	0.42857	0.53846	1.00000
E86	14	0.50000	0.42593	1.00000	--	--	--	--	13	0.15385	0.14769	1.00000
E90	14	0.35714	0.50529	0.00739	8	0.00000	0.23333	0.06690	13	0.00000	0.36923	0.00127
E97	14	0.78571	0.56878	0.17616	13	1.00000	0.63077	0.00484	11	0.45455	0.55844	0.04943
Ep05	15	0.73333	0.57011	0.41937	15	0.73333	0.66897	0.03284	13	0.53846	0.68615	0.09562
Ep61	15	0.73333	0.65287	0.09693	10	0.40000	0.67368	0.01272	9	0.66667	0.69935	0.00641
		Avg. Ho = 0.6468 Total Go = 114				Avg. Ho = 0.5835 Total Go = 87				Avg. Ho = 0.4148 Total Go = 92		
	HT for three populations = 0.54836											

Go—observed genotypes, Ho—observed heterozygosity, He—expected heterozygosity, HT—total heterozygosity.

**Table 4:** Analysis of molecular variance (AMOVA) using eight loci and three amplified microsatellites.

	With Eight Loci			With Three Loci		
Source of Variation	SC	CV	% VAR	SC	CV	% VAR
Between populations	10.956	0.12888	5.58728	5.371	0.05745	5.57
Between subjects within populations	78.049	0.01312	0.56864	41.687	0.06760	6.55
Within subjects	82.500	2.16472	93.84408	39.000	0.90698	87.88
Total	171.504	2.30672		86.058	1.03203	

SC: sum of squares, CV: variance components.

**Table 5:** Specific FIS indices by population.

Population	FIS	P (FIS ≥ FIS Observed)
1	−0.04103	0.691105
2	0.07109	0.276637
3	0.18710	0.069404

**Table 6:** FST pairwise comparison of populations using paired *t*-tests. Significance matrix FST (*p* = 0.05).

Population	1	2	3
1		+	+
2	+		-
3	+	-	

**Table 7:** Gene flow: matrix of M-values (M = 2 Nm).

	1	2	3
1			
2	6.16504		
3	4.26460	∞	

**Table 8:** The proportion of members of each predefined population in the two groups.

Population	Clusters		Number of Plants
	1	2	
1	0.634	0.366	15
2	0.453	0.547	15
3	0.447	0.553	13

**Table 9:** Variables used in the multi-criteria analysis to determine the potential distribution of *Euphorbia fulgens*.

Variable	Format	Scale/Resolution	Source
Temperature	TIF	1 km	UNAM, 2020
Precipitation	TIF	1 km	UNAM, 2020
Climate Units	Shapefile	1:250,000	UNAM, 2020
Altitude	TIF	15 m	INEGI, 2020
Vegetation type	Shapefile	1:250,000	INEGI, 2017
Slope	TIF	15 m	INEGI, 2020

## Data Availability

The datasets generated and/or analyzed during the current study are available from the corresponding author upon reasonable request.

## Supplementary Materials

The following supporting information can be downloaded at: https://www.mdpi.com/article/10.3390/plants11192542/s1, Table S1: Description of the microsatellite loci used in *Euphorbia fulgens*; S2: Methodology for soil analysis used at the Central University Laboratory. Soil Department. Universidad Autónoma Chapingo.
