# Supplementary material for: Diversity and Genetic Structure of Scarlet Plume (Euphorbia fulgens), an Endemic Plant of Mexico
Source: Plants (Basel). 2022 Sep 28;11(19):2542. doi: 10.3390/plants11192542 (PMC9572564; doi:10.3390/plants11192542)
Supplement: Supplementary file 1 [file plants-11-02542-s001.zip › plants-1911931-SI.pdf]

Supplementary material

Table S1. Description of the microsatellite loci used in *Euphorbia fulgens*.

Locus		Sequences →(5' 3')	Source	Number of alleles	TA (°C)
1	Ek3216	F: AAACCACAACTCAATTCA<br>R: AAGCAAAAGGACACAAAA	Yan *et al*., 2014	3	48
2	Ek8578	F: CAAAACCACAACCTTAAACCATCTA<br>R: TTCTTCCATTTCTGAAAATTGAGAG	Yan *et al*., 2014	3	42
3	E78	F: AGTGAGAAAGTGTGTGGATA<br>R: AGCCCACAGAAGCATAGCTC	Li *et al*., 2014	3	42
4	E86	F: CTCTGGGGCTTCTCCTGATA<br>R: ACACACCTGGACACACGGTA	Li *et al*., 2014	3	42
5	E90	F: CCATTCAAGCACGACTCAAC<br>R: AAGAGTTCAAGTGAGGGAAAG	Li *et al*., 2014	12	59
6	E92	F: AACACATGGCACCAGTGAAC<br>R: TTCCTTCTCTCTCTTCCATTCC	Li *et al*., 2014	3	--
7	E97	F: GAGAGATACAAGGAGCAAGCAA<br>R: TGACCCAATCTGCATACCAA	Li *et al*., 2014	4	48
8	Ep75	F: TGTACGCCTCTCTCTCACTCC<br>R: TGACGTCGAATTTGTAGTTGC	Durka, 2009	3	--
9	Ep05	F: AAAGCCCACTACGCAACAAG<br>R: AAAACACTCCGACGGTCAAG	Durka, 2009	6	59
10	Ep61	F: CAGATTCCAGAAAATCAACAGC<br>R: CGTCGTCTTCATTTCTGTCC	Durka, 2009	4	42

S2. Methodology for soil analysis used at the Central University Laboratory. Soil Department. Universidad Autónoma Chapingo

pH: Potentiometric, soil-water ratio, 1:2.

Electrical conductivity (EC): Electrical conductivity bridge in suspension sample: water, 1:2.

Organic Matter (OM): Wlakely and Black.

Total Nitrogen (N Tot.): Determined by steam entrainment: Kjeldahl.

Inorganic Nitrogen (N. Inorg.): Extracted with 2N potassium chloride and determined by vapor entrainment.

Assimilable Phosphorus (P): Bray P-1.

Potassium (K): Extracted in 1.0 N ammonium acetate, pH 7.0, ratio 1:20 and determined by flame emission spectrophotometry.

Calcium, Magnesium (Ca, Mg): Extracted with 1.0 N ammonium acetate, pH 7.0, ratio 1:20 and determined by atomic absorption spectrophotometry.

Iron, Copper, Zinc, Manganese (Fe, Cu, Zn, Mn): Extracted with DTPA ratio 1:4 and determined by atomic absorption spectrophotometry.

Boron (B): Extracted with CaCl2 1.0M and determined by photocolorimetry with Azomethine-H.

Bulk density (DBD): Test tube method.

Texture (Tex): Bouyoucos hydrometer.
